# Heat-Stable Hazelnut Profilin: Molecular Dynamics Simulations and Immunoinformatics Analysis

**DOI:** 10.3390/polym12081742

**Published:** 2020-08-05

**Authors:** Haruna L. Barazorda-Ccahuana, Vinicius Theiss-De-Rosso, Diego Ernesto Valencia, Badhin Gómez

**Affiliations:** 1 Centro de Investigación en Ingeniería Molecular—CIIM, Vicerrectorado de Investigación, Universidad Católica de Santa María, Urb. San José s/n—Umacollo, Arequipa 04000, Peru; dvalenciac@ucsm.edu.pe (D.E.V.); bgomez@ucsm.edu.pe (B.G.); 2 Setor de Ciências da Saúde, Universidade Federal do Paraná, Curitiba 80060-000, Brazil; rossovt@gmail.com

**Keywords:** hazelnut, molecular dynamics simulation, immunoinformatic, allergen, profilin

## Abstract

Heat treatment can modify the allergenic potential, reducing allergenicity in specific proteins. Profilins are one of the important hazelnut allergens; these proteins are considered panallergens due to their high capacity for cross-reactivity with other allergens. In the present work, we evaluated the thermostability of hazelnut profilin, combining molecular dynamics simulation and immunoinformatic techniques. This approach helped us to have reliable results in immunogenicity studies. We modeled Cor a 2 profilin and applied annealing simulation, equilibrium, and production simulation at constant temperatures ranging from 300 to 500 K using Gromacs software. Despite the hazelnut profilins being able to withstand temperatures of up to 400 K, this does not seem to reduce its allergenicity. We have found that profilin subjected to temperatures of 450 and 500 K could generate cross-reactivity with other food allergens. In conclusion, we note a remarkable thermostability of Cor a 2 at 400 K which avoids its structural unfolding.

## 1. Introduction

Hazelnut (*Corylus avellana*) is one of the most consumed foods in the world; it is an important supply of processed food and can be consumed raw or cooked [1]. Likewise, this tree nut is known for its health benefits [2,3,4,5,6,7].

Hazelnut consumption can also cause allergic reactions, generating mild contact symptoms up to life-threatening systemic hypersensitivity reactions [8,9,10]. Hazelnut has 9 identified allergens: Cor a 1 (PR-10 protein), Cor a 2 (profilin), Cor a 8 (non-specific lipid transfer protein), Cor a 9 (seed legumin), Cor a 10 (pollen protein), Cor a 11 (vicilin), ref [11] Cor a 12 (Oleosin), Cor a 13 (Oleosin), and Cor a 14 (albumin) [12]. Cor a 2 has a molecular weight of 14 kDa, ref [13] and plays an important role in the cytoskeletal organization’s process, joining actin filaments [14]. Although hazelnut profilin is considered a minor allergen, it has clinical relevance evidenced in several studies, which is considered more important than the Cor a 1 allergen [15,16].

The influence of heat treatment on food processing has an impact on the allergenic and antigenic integrity of tree nut extracts [17]. The heat-resistant allergenic potential of hazelnut above 413.15 K by in vitro assays has been analyzed by many researchers, finding the allergenic potential of hazelnut at high temperatures is affected, demonstrated by decreased IgE binding [18,19]. Additionally, M. Wigotzki et al. found that proteins with low weight are stable at even higher temperatures [19].

The thermostability of proteins has significant repercussions in the properties of their native folded structure. Furthermore, these proteins have many individual factors achieving stability at high temperatures [20]. This behavior can be attributed to hydrogen bonding, salt bridges, van der Waals’ interactions, hydrophobic internal packing, improved electrostatic interactions, and others [21,22]. Currently, molecular dynamics simulation techniques allow us to understand the behavior of many proteins submitted to high temperatures, and to enable us to predict their behavior [23,24,25,26]. For example, the soy allergen has been analyzed by molecular dynamics simulations methods to understand the structural conformation for the development of antiallergenic drugs [27,28]. Another interesting case is the study of the peanut conglutin, to understand the overall conformational stability of the protein and their IgE binding epitopes [29].

The present research focused on analyzing the impact of temperature on the allergenicity of hazelnut profilin through molecular dynamics simulations. Additionally, immunoinformatic tools were used to predict linear epitopes and cross-reactivity, among other food allergens.

## 2. Materials and Methods

### 2.1. Protein Preparation

Cor a 2 profilin sequence (GenBank access code: AAK01236.1) was obtained from NCBI (National Center of Biotechnology Information) database. We have used Swiss-Model server (http://swissmodel.expasy.org/) [30] for the homology modeling. We considered the template based on the best resolution and its sequence identity percentage [31]. PDB2PQR Server (http://nbcr-222.ucsd.edu/pdb2pqr_2.0.0/) determined the correct protonation states of the amino acids at pH 7.

### 2.2. Molecular Dynamics Simulation

The molecular dynamics (MD) simulations were performed in Gromacs 2019, ref [32] and we considered OPLS-AA force field. OPLS-AA is an interesting force field that considers all atoms of a protein explicitly and is useful in the study of the organic and biomolecular systems [33]. The protein was situated inside a cubic box with a distance of 1.5 nm to the edges of the box and the explicit TIP4P [34] water model was considered. Na${}^{+}$ ions were added to neutralize the system. The energy minimization using steepest descent method of 200,000 steps with 0.001 nm initial step-size was employed.

Leap-frog algorithm for integrating Newton’s equations was employed to MD simulations. The bond lengths were constrained by the LINCS (LINear Constraint Solver) algorithm, ref [35] and periodic boundary conditions (PBC) in all directions, were used. Cut-off of 0.9 nm distance for the short-range interactions and Particle-Mesh Ewald (PME) [36] method for the long-range interactions were considered. V-rescale thermostat and Parrinello-Rahman barostat regulated the temperature (300 K) and pressure (1 bar).

We applied three MD steps: annealing, equilibrium, and production simulation. The first, simulated annealing procedure consisted of 20 annealing cycles in 500 ps with a high temperature of 400 K and a cooling temperature until 300 K by 10 ns. In the second step, the equilibration was performed in the NVT (number of molecules, volume, and temperature constant) canonical ensemble by a short time of 10 ns for integration, considering position restraint. They were restrained with harmonic forces and the water molecules were allowed to equilibrate around the protein. In the third step, we considered the MD in the isobaric-isothermal ensemble without position restraint during 200 ns was calculated. Furthermore, five systems with these temperatures: 300 K, 350 K, 400 K, 450 K, and 500 K, were considered at this point.

The analysis of Root-mean-square deviation (RMSD), Root-mean-square fluctuation (RMSF), radii of gyration (Rg), Solvent Accessibility Surface Area (SASA), and hydrogen bond (Hb), were obtained using the GROMACS tools and plotted in Gnuplot 5.0 software. We considered the average values of the last 20 ns of the MD simulation. Salt bridges of 5 frames of the trajectory (180 nm, 185 nm, 190 nm, 195 nm, and 200 nm) were analyzed by VMD (Visual Molecular Dynamics) [37] software. All graphics representation was recreated in Chimera UCSF 11.1.2 software [38].

### 2.3. Immunoinformatic Details

The last frame of MD simulations was analyzed through Ellipro server (http://tools.iedb.org/ellipro/). The server predicts linear and discontinuous antibody epitopes based on a three-dimensional structure protein antigen [39]. We have considered the minimum score of 0.5 and the maximum distance of 6 Angstroms.

Identification of potential cross-reactive Cor a 2, was predicted by Cross-React server (http://curie.utmb.edu/Cross-React.html), a tool that uses the epitope location and the three-dimensional structure of protein allergens [40]. A patch size (A) of 10, an area cutoff (A${}^{2}$) of 10, and the correlation cutoff of 0.10, were applied.

## 3. Results and Discussion

**Figure 1:**
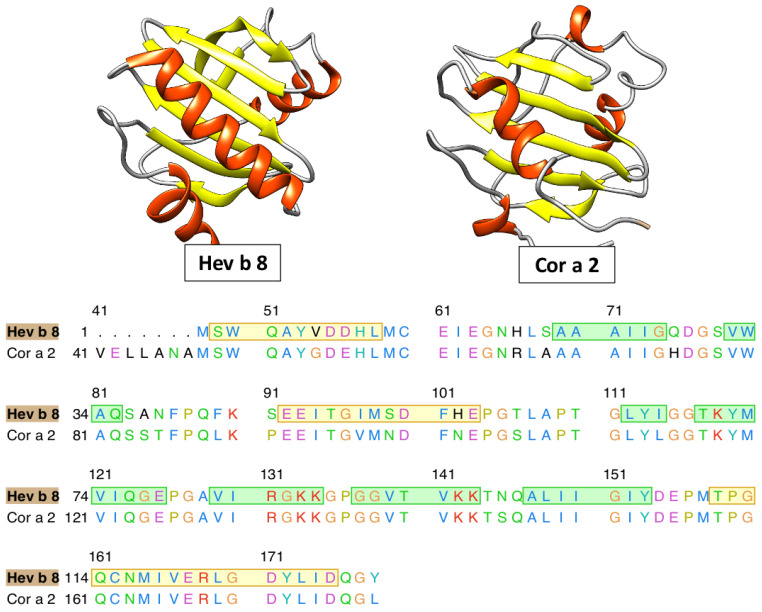
Ribbon schematic representation of profilins and multiple alignments with 88.4% of identity between them.

Currently, the three-dimensional structures of all profilins have not been entirely reconstructed; consequently, we have to use alternative methods to study non-crystallized proteins. This study was based on the clinical relevance of hazelnut allergenicity, particularly focused on profilin (Cor a 2). We have identified Hev b 8 as an adequate template (PDB ID: 5FDS), ref [41] which has a 1.9 Å resolution (resolved by X-ray diffraction) and a sequence identity percentage of 88.46% with Cor a 2 (see Figure 1).

### 3.1. MD Simulation Analysis

**Figure 2:**
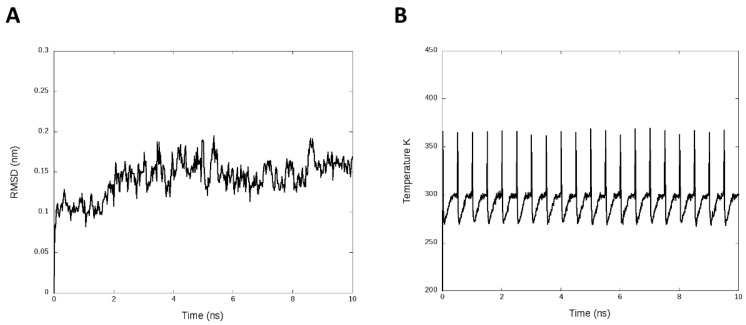
Simulated annealing analysis. (**A**) Root-mean-square deviation (RMSD) by 10 ns of the annealing simulation. (**B**) Temperature plot with the cycles generates along the MD annealing simulation.

We introduced simulated annealing. This analysis refined and fitted the three-dimensional structure of protein after the prediction of homology modeling [42]. We used the periodic cycles of 400 K, 280 K, 270 K, 298 K, and 300 K for 0 ps, 30 ps, 60 ps, 300 ps, and 500 ps, respectively. Figure 2A presents the RMSD plot with an average of 0.14 ± 0.02 nm, and Figure 2B shows the temperature cycles during the simulation.

For the MD calculus, we have used a OPLS-AA force field characterized by calculating the conformational energy of organic and biomolecular systems. This force field allows us to reproduce the experimental properties of the condensed structural and thermodynamic phases [43,44].

Furthermore, the selection of the water model plays an important role in these types of simulations, in which we look at the behavior at temperatures above 300 K. The best water model chosen for the study was the four-site water model TIP4P [45]; this water model is capable of inducing the values of physical properties and achieves a better approximation [46,47,48].

**Figure 3:**
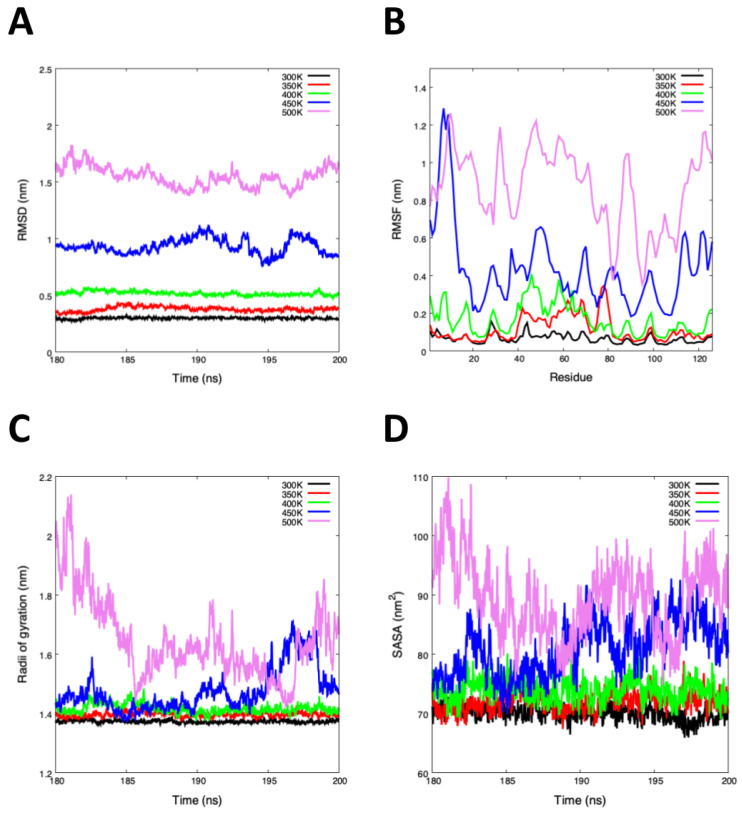
MD simulation analysis of the last 20 ns. (**A**) Root-mean-square deviation (RMSD) showing the high structural instability of systems between 450 K and 500 K. (**B**) Root-mean-square fluctuation (RMSF) plot show high fluctuations at 450 K and 500 K, this analysis was based on the C-α. (**C**) Radii of gyration (Rg) plot shows us the high compactness between 300 to 400 K. (**D**) Solvent Accessibility Surface Area (SASA) plot indicated that exists a large surface area at high temperatures (450 K and 500 K).

Table 1 and Figure 3 show the average of RMSD, RMSF, Rg, SASA, and Hb of the last 20 ns. The RMSD average shows an increase from 300 to 500 K, the lowest and highest values were indicated as high and low stability, respectively (Figure 3A). While the average RMSF values show an abrupt increase at 450 K and 500 K, the high flexibility values were found in the middle of the structure, with the loss of the beta-strands, provoking the unfolding process (Figure 3B). While the Rg value at 500 K was higher than 300 K, there is less structural compaction at 450 K and 500 K (Figure 3C).

An interesting result of the molecular dynamics simulation was the analysis of the solvent-accessible surface area (SASA). Generally, SASA defines the surface around a protein model calculated based on the study of the hypothetical center of a water sphere (approximately one molecule of water) [49]. The total SASA values increased from 69.96 to 89.40 nm${}^{2}$ with the temperature change, suggesting that the total hydrophobicity of Cor a 2 was reduced generating conformational changes (Figure 3D). The low variation in Cor a 2 at 300 K, 350 K, and 400 K denotes higher thermodynamic stability than 450 K and 500 K. In Table 1 the Hb analysis showed us that the highest temperatures (450 K and 500 K) led to the loss of secondary structure conformation due to the reduction of their hydrogen bonds.

The analysis of salt bridges in macromolecules allows describing of the structure and function of proteins. Usually, these occur between negative (ASP and GLU) and positive (LYS and ARG) amino acids. In this study, the analysis of five frames of the last 20 ns, was performed. We analyzed the salt bridges of proteins, where the 450 K and 500 K systems had the highest salt bridges (See Table 2). Adrian Helcock in 1998 proposed that salt bridges play a crucial role in promoting hyperstability in proteins; however, they seem to contribute low stability of the protein at room temperature [50]. Therefore, the increase in salt bridges at high temperatures may imply a greater resistance to Cor a 2 denaturation.

**Figure 4:**
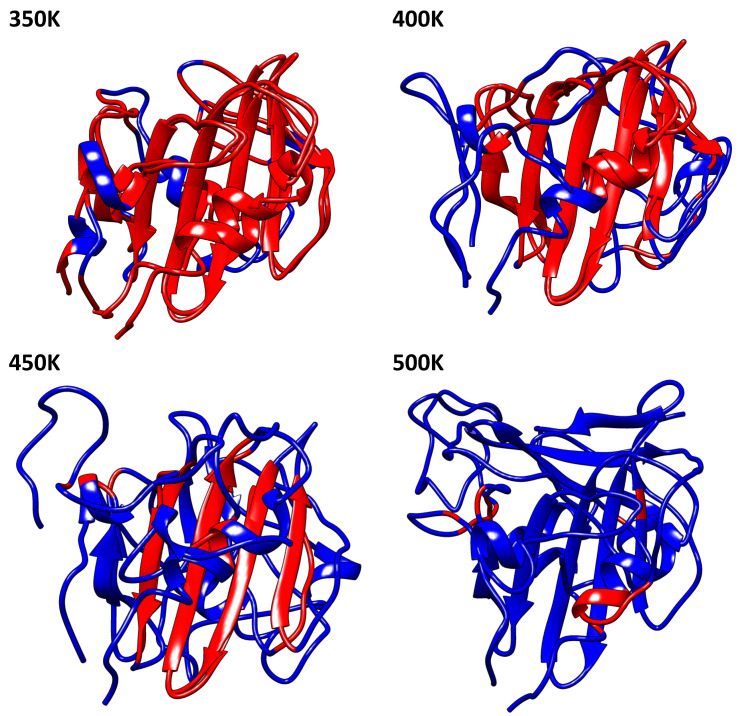
Molecular structure comparison of 300 K between 350 K, 400 K, 450 K, and 500 K, the red color show us the structural conservation. For additional information of beta-sheet conformation see Table S6 of supplementary material.

The presence of β-sheets in the conformational structure of profilins define its high stability. Indeed, the β-sheets seem to be the most important region of the thermostability in these proteins. Figure 4 shows us in red the structure conserved concerning the protein at 300 K. Profilin at 350 K maintained the major regions of 300 K, unlike 400 K and 450 K in which only the motif of β-sheets is conserved. The secondary structure of the profilin at different temperatures showed us slight, but relevant, variations in their conformation. We observed quantitative variations of β-sheets conformation at each temperature; for 300 K, 350 K, 400 K, 450 K, and 500 K the percentage were 26.2%, 25.4%, 26.2%, 21.5%, and 15.4% respectively. A tendency to decrease the number of residues that form the β-sheets was observed; it is directly linked to the protein denaturation due to the increased temperature (For additional information see Table S6).

**Figure 5:**
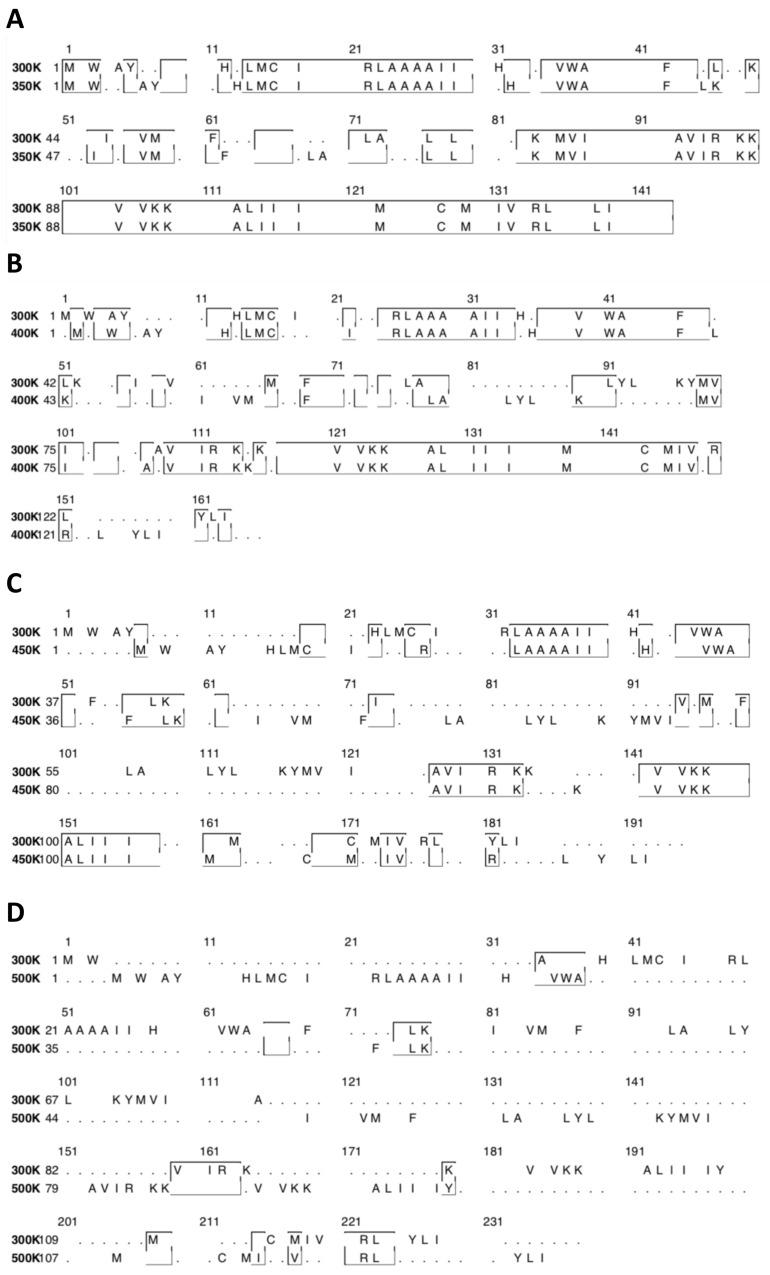
The alignment sequence of molecular structure was analyzed at a different temperature, with 300 K as a reference temperature. The box shows us the sequence conservation at different temperature as indicated by the letters (**A**–**D**).

Another point of view is the sequence conservation, Figure 5 shows the sequence alignment between 300 K and the other temperatures analyzed. We identify in the box the structural conservation for each system.

### 3.2. Immunoinformatics

The antigenic B cell epitopes prediction has clinical importance because it helps to identify areas of an antigen recognized by antibodies [51]. In this work, we used the Ellipro server, which is a handy immunoinformatic tool based on three algorithms. The first is the approximation of the protein shape as an ellipsoid. The second approximation is the residue protrusion index (PI). The third is the clustering of neighboring residues based on their PI values. These criteria allow analyzing linear and discontinuous epitopes of a protein [52,53].

For this analysis, we have used the last frame at each temperature. The Table 3 describes the score and the number of residues for each linear epitope. These results show that epitopes are reduced when the system is studied at 500 K. Additional information is shown in Tables S1–S5, in which we show the analysis of the epitopes of five frames of the last 20 ns. However, despite a reduction of epitopes, several allergenic regions persist in all cases.

In order to complement the allergenicity study, we performed the cross-reactivity analysis, based on the epitopes of the last frame. For this case, we have considered a Pearson correlation coefficient (PCC) with a cut-off of 0.8. The results show us cross-reactivity of Cor a 2 at 300 K with 24 allergens from different plants and a decrease in cross-reactivity at 500 K.

We analyzed the percentage of intensity following the Equation (1), where; $E_{C-R}$ is the number of epitopes with a PPC greater than 0.8, a product of cross-reactivity analysis (cross-reactivity epitopes), and $E_{n}$ is the number of epitopes (linear epitopes) by Cor a 2 at different temperatures.

(1) $$\frac{E_{C-R}}{E_{n}}.100\%=Intensity\%$$

**Figure 6:**
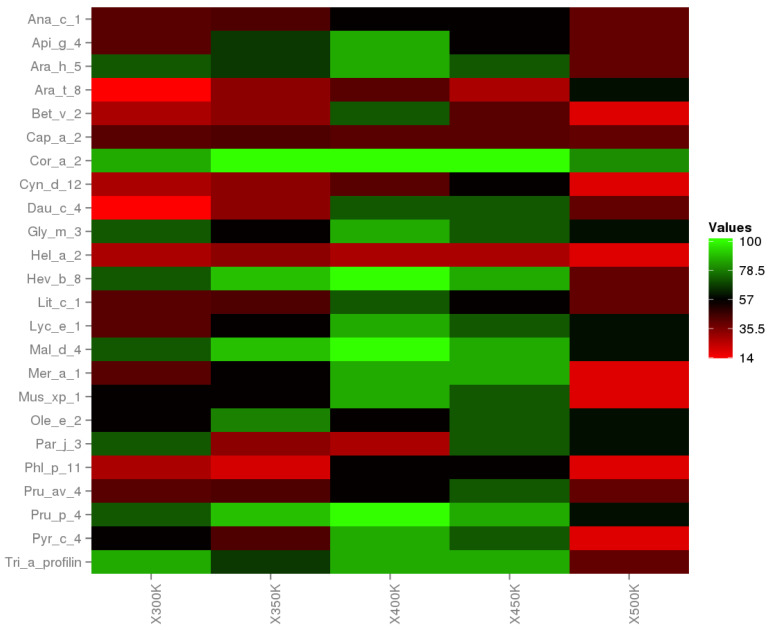
Heat plot. The cross-reactivity of Cor a 2 and 24 allergens are representants by the percentage of predominance; where 100% (green color) indicates that all epitopes are shown for a particular allergen and a given temperature.

Linear epitopes and cross-reactivity of Cor a 2 were evaluated through temperature changes, showing significant variation. The heat map (Figure 6) exhibits the intensity percentage of these variations where the green color means hight cross-reactivity, and the red color implies the lost cross-reactivity. In Supplementary Information, Figures S1–S5 show in detail the cross-reactivity prediction for each system. Moreover, the major cross-reactivity occurs between 350 K, 400 K, and 450 K, and the effect is reduced at 500 K. However, Cor a 2 at 500 K has a 50% prevalence that generates cross-reactivity with other allergens. With this last prediction, we show that hazelnut profilin at temperatures among 300 K and 500 K generates cross-reactivity with other food allergens.

## 4. Conclusions

In this research, we have performed molecular dynamics simulations at constant temperatures of 300 K, 350 K, 400 K, 450 K, and 500 K by 200 ns. These methods allowed us to understand the remarkable thermostability of Cor a 2 at temperatures up to 400 K. The salt bridges played an important role in the thermoresistance analysis of profilin. We saw that these salt bridges might avoid the complete denaturation of Cor a 2 at high temperatures as well as the case of thermophilic proteins.

Moreover, the analysis of the number of linear epitopes based on 3D structures, demonstrated that there is a reduction of epitopes at temperatures of 500 K. However, the decrease in epitopes of Cor a 2 at 500 K does not show the complete loss of its allergenicity. Finally, the cross-reactivity prediction identified many potential allergens that could generate cross-reactivity with Cor a 2 at high temperatures.

## Figures and Tables

**Table 1:** Summary of RMSD, RMSF, Rg, SASA, and Hb average values of the last 20 ns.

Temperature	RMSD ${}^{a}$	RMSF ${}^{a}$	Rg ${}^{a}$	SASA ${}^{b}$	Hb ${}^{c}$
300 K	0.30 ± 0.01	0.07 ± 0.03	1.37 ± 0.01	69.96 ± 1.24	83 ± 4.4
350 K	0.38 ± 0.02	0.11 ± 0.07	1.39 ± 0.01	71.99 ± 1.69	81 ± 4.5
400 K	0.51 ± 0.02	0.17 ± 0.09	1.40 ± 0.02	73.88 ± 1.95	74 ± 4.9
450 K	0.93 ± 0.07	0.44 ± 0.22	1.45 ± 0.04	80.87 ± 4.67	69 ± 6.7
500 K	1.55 ± 0.09	0.86 ± 0.21	1.61 ± 0.13	89.40 ± 6.31	70 ± 8.3

^*a*^ Values are in nm; ^*b*^ Value of SASA is in nm${}^{2}$; ^*c*^ Number of hydrogen bond.

**Table 2:** Salt bridges (SB) analysis of each temperature.

Frame	300 K		350 K		400 K		450 K		500 K	
	SB	d	SB	d	SB	d	SB	d	SB	d
**180 ns**	GLU108-LYS86	2.6	GLU46-LYS43	2.4	GLU45-LYS43	2.7	GLU108-LYS86	2.6	ASP29-LYS96	3.4
	GLU78-ARG84	4.6	GLU9-ARG19	4.1	GLU16-ARG19	3.6	ASP107-ARG19	4.6	GLU108-LYS86	3.6
	GLU120-LYS95	2.7	ASP53-LYS96	3.5	GLU120-LYS95	2.8	ASP128-ARG121	4.1	GLU56-HIS28	4.5
	GLU46-LYS43	3.4	GLU16-ARG121	4.6	GLU78-ARG84	4.0	GLU45-LYS43	3.6	GLU45-LYS43	3.0
	GLU9-ARG19	4.4	GLU78-ARG84	3.3	GLU14-ARG19	3.3	GLU56-LYS96	2.8	GLU46-LYS71	3.5
					ASP53-LYS96	3.2	ASP124-ARG121	4.4	GLU120-LYS86	3.9
					GLU56-LYS96	2.6	GLU56-HIS28	4.8	GLU16-LYS95	3.4
					ASP53-HIS28	4.8	GLU46-LYS87	3.1	GLU78-LYS96	3.5
					GLU108-ARG84	3.9				
**185 ns**	GLU108-LYS86	3.1	GLU108-ARG84	3.8	GLU45-LYS43	2.7	ASP53-ARG84	3.2	GLU45-LYS71	3.1
	GLU46-LYS43	2.7	GLU9-ARG19	4.4	GLU108-ARG84	4.0	GLU45-LYS43	3.2	GLU108-ARG84	3.3
	GLU9-ARG19	4.3	GLU46-LYS43	3.1	GLU120-LYS95	3.1	ASP107-LYS71	2.9	GLU45-LYS43	3.6
	GLU120-LYS95	3.0	ASP53-LYS96	3.7	GLU78-ARG84	4.0	GLU56-LYS96	3.7	GLU56-LYS43	3.4
					GLU56-LYS96	3.0	GLU108-LYS86	3.2	ASP128-LYS86	3.8
					ASP107-ARG19	4.7	GLU78-ARG84	3.7	ASP53-LYS71	3.3
					GLU14-ARG19	3.2	ASP8-HIS10	4.1	GLU78-LYS96	2.5
					ASP124-ARG121	4.5	ASP107-ARG19	4.5	GLU78-ARG121	4.4
					GLU9-HIS10	3.8	GLU56-HIS28	4.5	ASP128-LYS87	3.4
					GLU16-ARG19	3.4			GLU120-LYS87	3.2
**190 ns**	GLU108-LYS86	2.6	GLU16-ARG121	4.7	GLU45-LYS43	3.3	ASP107-LYS71	2.5	ASP128-LYS87	3.3
	GLU9-ARG19	4.2	GLU46-LYS43	2.7	GLU108-ARG84	3.8	ASP128-ARG19	3.6	GLU78-ARG121	3.5
	GLU78-ARG84	4.4	GLU9-ARG19	5.0	GLU108-LYS86	3.5	GLU78-ARG84	3.5	ASP128-LYS86	3.0
	GLU120-LYS95	3.0	ASP53-LYS96	3.6	GLU120-LYS95	2.7	ASP53-ARG84	4.2	GLU14-LYS95	2.9
			GLU108-ARG84	3.6	GLU78-ARG84	4.3	GLU108-LYS86	3.5	GLU46-LYS71	2.7
					GLU16-ARG19	3.5	GLU78-LYS87	2.9	GLU9-HIS10	3.9
					GLU14-ARG19	3.3	GLU16-ARG121	3.3	GLU120-LYS86	3.1
					ASP124-ARG121	4.8			GLU120-LYS87	3.1
									GLU78-LYS96	3.6
**195 ns**	GLU108-LYS86	2.7	GLU9-ARG19	3.4	GLU45-LYS43	3.4	GLU120-ARG121	3.3	GLU9-LYS71	2.8
	GLU9-ARG19	4.5	GLU108-LYS86	3.4	ASP124-ARG121	3.5	GLU78-LYS87	3.6	GLU120-LYS87	3.1
	GLU78-ARG84	4.5	GLU46-LYS43	3.1	GLU108-ARG84	4.2	ASP107-LYS71	3.1	ASP128-LYS87	3.3
	GLU120-LYS95	3.4	ASP53-LYS96	3.7	GLU78-ARG84	4.2	GLU56-LYS96	3.1	ASP29-LYS96	4.1
			GLU108-ARG84	4.3	GLU14-ARG19	3.3	GLU108-LYS86	3.8	GLU16-ARG19	4.1
					ASP53-LYS96	3.7	ASP128-ARG19	3.6	ASP128-LYS86	3.1
					GLU9-HIS10	4.4	GLU56-HIS28	4.5	GLU46-LYS43	3.7
					GLU16-ARG19	3.2	ASP124-ARG19	3.6	ASP8-LYS95	3.3
					GLU120-LYS95	3.2	GLU78-ARG84	4.4	ASP124-ARG121	4.3
									GLU120-LYS86	2.9
									GLU78-LYS96	3.9
**200 ns**	GLU108-LYS86	2.7	GLU46-LYS43	2.6	GLU78-ARG84	4.4	GLU78-ARG84	3.3	GLU120-LYS87	2.5
	GLU9-ARG19	4.4	ASP53-LYS96	3.4	GLU14-ARG19	3.3	ASP107-LYS71	3.2	ASP29-LYS96	2.5
	GLU120-LYS95	3.0	GLU108-ARG84	3.9	GLU108-LYS86	3.3	GLU108-LYS86	3.1	ASP8-LYS95	2.9
					GLU45-LYS43	2.7	GLU45-LYS43	3.6	GLU45-LYS43	3.3
					ASP53-LYS96	3.6	ASP53-LYS43	2.5	ASP128-LYS86	3.5
					GLU16-ARG19	3.3	GLU56-LYS43	3.3	GLU14-LYS71	3.2
					GLU108-ARG84	4.1	ASP124-ARG19	3.5		
					GLU120-LYS95	3.3	GLU120-LYS95	3.4		

d: distance in Å between atoms of basic and acidic residues.

**Table 3:** Epitope prediction of the last frame of MD simulation at different temperatures.

Start	End	Peptide	N.residues	Score *
**300 K**				
124	130	DYLIDQG	7	0.7
40	46	PQLKPEE	7	0.7
1	10	MSWQAYGDEH	10	0.7
87	90	KGPG	4	0.6
107	115	DEPMTPGQC	9	0.6
51	81	MNDFNEPGSLAPTGLYLGGTKYMVIQGEPGA	31	0.6
13	20	CEIEGNRL	8	0.6
**350 K**				
40	47	PQLKPEEI	8	0.7
52	64	NDFNEPGSLAPTG	13	0.7
1	10	MSWQAYGDEH	10	0.7
107	117	DEPMTPGQCNM	11	0.7
86	89	KKGP	4	0.6
123	130	GDYLIDQG	8	0.6
68	81	GGTKYMVIQGEPGA	14	0.6
13	20	CEIEGNRL	8	0.6
27	31	GHDGS	5	0.5
**400 K**				
123	130	GDYLIDQG	8	0.8
108	114	EPMTPGQ	7	0.7
44	64	PEEITGVMNDFNEPGSLAPTG	21	0.6
76	81	QGEPGA	6	0.6
1	19	MSWQAYGDEHLMCEIEGNR	19	0.6
28	33	HDGSVW	6	0.6
69	73	GTKYM	5	0.5
**450 K**				
125	130	YLIDQG	6	0.8
68	72	GGTKY	5	0.7
1	17	MSWQAYGDEHLMCEIEG	17	0.7
41	60	QLKPEEITGVMNDFNEPGSL	20	0.7
107	117	DEPMTPGQCNM	11	0.6
27	30	GHDG	4	0.6
78	89	EPGAVIRGKKGP	12	0.5
**500 K**				
1	7	MSWQAYG	7	0.8
47	66	ITGVMNDFNEPGSLAPTGLY	20	0.8
107	126	DEPMTPGQCNMIVERLGDYL	20	0.7
29	40	DGSVWAQSSTFP	12	0.6
76	80	QGEPG	5	0.5

* The score value is defined as a PI value averaged over epitope residues.

## Supplementary Materials

The following are available online at https://www.mdpi.com/2073-4360/12/8/1742/s1.
