# Supplementary material for: Heat-Stable Hazelnut Profilin: Molecular Dynamics Simulations and Immunoinformatics Analysis
Source: Polymers (Basel). 2020 Aug 5;12(8):1742. doi: 10.3390/polym12081742 (PMC7464029; doi:10.3390/polym12081742)
Supplement: Supplementary file 1 [file polymers-12-01742-s001.pdf]

Supplementary Materials: Heat-Stable Hazelnut Profilin: Molecular Dynamics Simulations and Immunoinformatics Analysis

Haruna L. Barazorda-Ccahuana [1,‡*], Vinicius Theiss-De-Rosso [2,‡], Diego Ernesto Valencia [1,‡] and Badhin Gómez [1,]

Table S1. Epitopes prediction of the Cor a 2 at 300 K

Start	End	Peptide	Number of residues	score
		**t = 180 ns**		
124	130	DYLIDQG	7	0.731
107	114	DEPMTPGQ	8	0.679
87	90	KGPG	4	0.638
76	81	QGEPGA	6	0.629
1	11	MSWQAYGDEHL	11	0.619
13	20	CEIEGNRL	8	0.602
51	73	MNDFNEPGSLAPTGLYLGGTKYM	23	0.599
27	33	GHDGSVW	7	0.552
		**t = 185 ns**		
124	130	DYLIDQG	7	0.763
107	114	DEPMTPGQ	8	0.651
1	11	MSWQAYGDEHL	11	0.643
55	73	NEPGSLAPTGLYLGGTKYM	19	0.639
76	81	QGEPGA	6	0.624
40	46	PQLKPEE	7	0.62
87	90	KGPG	4	0.617
13	20	CEIEGNRL	8	0.606
		**t = 190 ns**		
123	130	GDYLIDQG	8	0.688
107	114	DEPMTPGQ	8	0.665
1	10	MSWQAYGDEH	10	0.649
87	90	KGPG	4	0.633
52	73	NDFNEPGSLAPTGLYLGGTKYM	22	0.625
76	81	QGEPGA	6	0.596
13	20	CEIEGNRL	8	0.592
40	46	PQLKPEE	7	0.591
27	34	GHDGSVWA	8	0.556
		**t = 195 ns**		
124	130	DYLIDQG	7	0.742
40	46	PQLKPEE	7	0.678
87	90	KGPG	4	0.637
107	115	DEPMTPGQC	9	0.624
1	11	MSWQAYGDEHL	11	0.61
13	20	CEIEGNRL	8	0.6
51	73	MNDFNEPGSLAPTGLYLGGTKYM	23	0.598
76	81	QGEPGA	6	0.59
		**t = 200 ns**		
124	130	DYLIDQG	7	0.72
40	46	PQLKPEE	7	0.676
1	10	MSWQAYGDEH	10	0.66
87	90	KGPG	4	0.633
107	115	DEPMTPGQC	9	0.626
51	81	MNDFNEPGSLAPTGLYLGGTKYMVIQGEPGA	31	0.6
13	20	CEIEGNRL	8	0.563

Table S2. Epitopes prediction of the Cor a 2 at 350 K

Start	End	Peptide	Number of residues	score
		**t = 180 ns**		
68	81	GGTKYMVIQGEPGA	14	0.514
27	32	GHDGSV	6	0.551
13	19	CEIEGNR	7	0.579
87	90	KGPG	4	0.621
51	65	MNDFNEPGSLAPTGL	15	0.629
40	49	PQLKPEEITG	10	0.664
1	10	MSWQAYGDEH	10	0.669
107	117	DEPMTPGQCNM	11	0.701
125	130	YLIDQG	6	0.737
		**t = 185 ns**		
126	130	LIDQG	5	0.757
108	117	EPMTPGQCNM	10	0.728
75	81	IQGEPGA	7	0.699
1	10	MSWQAYGDEH	10	0.676
50	60	VMNDFNEPGSL	11	0.674
40	47	PQLKPEEI	8	0.643
13	18	CEIEGN	6	0.599
66	73	YLGGTKYM	8	0.547
27	31	GHDGS	5	0.529
		**t = 190 ns**		
126	130	LIDQG	5	0.786
108	117	EPMTPGQCNM	10	0.716
51	60	MNDFNEPGSL	10	0.678
40	47	PQLKPEEI	8	0.664
1	11	MSWQAYGDEHL	11	0.648
13	19	CEIEGNR	7	0.579
62	81	PTGLYLGGTKYMVIQGEPGA	20	0.541
		**t = 195 ns**		
54	59	FNEPGS	6	0.758
76	81	QGEPGA	6	0.738
108	117	EPMTPGQCNM	10	0.721
1	9	MSWQAYGDE	9	0.706
40	47	PQLKPEEI	8	0.684
123	130	GDYLIDQG	8	0.616
13	18	CEIEGN	6	0.59
27	31	GHDGS	5	0.537
62	73	PTGLYLGGTKYM	12	0.517
		**t = 200 ns**		
40	47	PQLKPEEI	8	0.738
52	64	NDFNEPGSLAPTG	13	0.669
1	10	MSWQAYGDEH	10	0.667
107	117	DEPMTPGQCNM	11	0.666
86	89	KKGP	4	0.644
123	130	GDYLIDQG	8	0.6
68	81	GGTKYMVIQGEPGA	14	0.557
13	20	CEIEGNRL	8	0.554

27	31	GHDGS	5	0.534

Table S3. Epitopes prediction of the Cor a 2 at 400 K

Start	End	Peptide	Number of residues	score
		**t = 180 ns**		
123	130	GDYLIDQG	8	0.785
1	11	MSWQAYGDEHL	11	0.685
36	52	SSTFPQLKPEEITGVMN	17	0.657
55	63	NEPGSLAPT	9	0.656
28	33	HDGSVW	6	0.608
108	115	EPMTPGQC	8	0.563
96	99	KTSQ	4	0.544
13	20	CEIEGNRL	8	0.523
		**t = 185 ns**		
40	52	PQLKPEEITGVMN	13	0.767
120	130	ERLGDYLIDQG	11	0.684
54	63	FNEPGSLAPT	10	0.67
1	10	MSWQAYGDEH	10	0.667
86	89	KKGP	4	0.623
108	114	EPMTPGQ	7	0.604
75	81	IQGEPGA	7	0.59
13	18	CEIEGN	6	0.587
		**t = 190 ns**		
123	130	GDYLIDQG	8	0.778
44	64	PEEITGVMNDFNEPGSLAPTG	21	0.722
1	11	MSWQAYGDEHL	11	0.647
107	114	DEPMTPGQ	8	0.626
76	81	QGEPGA	6	0.571
13	19	CEIEGNR	7	0.543
		**t = 195 ns**		
123	130	GDYLIDQG	8	0.805
1	10	MSWQAYGDEH	10	0.671
44	65	PEEITGVMNDFNEPGSLAPTGL	22	0.667
27	33	GHDGSVW	7	0.602
97	100	TSQA	4	0.594
108	115	EPMTPGQC	8	0.588
13	18	CEIEGN	6	0.577
		**t = 200 ns**		
123	130	GDYLIDQG	8	0.792
108	114	EPMTPGQ	7	0.662
44	64	PEEITGVMNDFNEPGSLAPTG	21	0.649
76	81	QGEPGA	6	0.64
1	19	MSWQAYGDEHLMCEIEGNR	19	0.613
28	33	HDGSVW	6	0.556
69	73	GTKYM	5	0.526

Table S4. Epitopes prediction of the Cor a 2 at 450 K

Start	End	Peptide	Number of residues	score
		**t = 180 ns**		
65	72	LYLGGTKY	8	0.827
4	19	QAYGDEHLMCEIEGNR	16	0.682
119	130	VERLGDYLIDQG	12	0.672
96	100	KTSQA	5	0.671
27	33	GHDGSVW	7	0.611
52	59	NDFNEPGS	8	0.604
42	49	LKPEEITG	8	0.6
		**t = 185 ns**		
65	72	LYLGGTKY	8	0.755
27	30	GHDG	4	0.688
51	58	MNDFNEPG	8	0.675
1	19	MSWQAYGDEHLMCEIEGNR	19	0.655
107	115	DEPMTPGQC	9	0.62
44	49	PEEITG	6	0.613
117	127	MIVERLGDYLI	11	0.599
		**t = 190 ns**		
119	130	VERLGDYLIDQG	12	0.772
95	100	KKTSQA	6	0.74
27	30	GHDG	4	0.692
1	15	MSWQAYGDEHLMCEI	15	0.636
38	58	TFPQLKPEEITGVMNDFNEPG	21	0.616
107	115	DEPMTPGQC	9	0.596
		**t = 195 ns**		
125	130	YLIDQG	6	0.745
1	17	MSWQAYGDEHLMCEIEG	17	0.738
68	72	GGTKY	5	0.689
106	116	YDEPMTPGQCN	11	0.667
44	61	PEEITGVMNDFNEPGSLA	18	0.665
		**t = 200 ns**		
125	130	YLIDQG	6	0.837
68	72	GGTKY	5	0.717
1	17	MSWQAYGDEHLMCEIEG	17	0.7
41	60	QLKPEEITGVMNDFNEPGSL	20	0.657
107	117	DEPMTPGQCNM	11	0.612
27	30	GHDG	4	0.575
78	89	EPGAVIRGKKGP	12	0.524

Table S5. Epitopes prediction of the Cor a 2 at 500 K

Start	End	Peptide	Number of residues	score
		**t = 180 ns**		
110	128	MTPGQCNMIVERLGDYLID	19	0.745
1	10	MSWQAYGDEH	10	0.704
58	80	GSLAPTGLYLGGTKYMVIQGEPG	23	0.678
29	34	DGSVWA	6	0.591
42	55	LKPEEITGVMNDFN	14	0.591
87	90	KGPG	4	0.563
		**t = 185 ns**		
62	69	PTGLYLGG	8	0.729
41	58	QLKPEEITGVMNDFNEPG	18	0.691
1	21	MSWQAYGDEHLMCEIEGNRLA	21	0.667
85	92	GKKGPGGV	8	0.652
104	127	GIYDEPMTPGQCNMIVERLGDYLI	24	0.589
		**t = 190 ns**		
40	60	PQLKPEEITGVMNDFNEPGSL	21	0.701
1	21	MSWQAYGDEHLMCEIEGNRLA	21	0.691
84	92	RGKKGPGGV	9	0.597
65	72	LYLGGTKY	8	0.57
104	127	GIYDEPMTPGQCNMIVERLGDYLI	24 0.55	
		**t = 195 ns**		
11	20	LMCEIEGNRL	10	0.724
38	65	TFPQLKPEEITGVMNDFNEPGSLAPTGL	28	0.697
106	127	YDEPMTPGQCNMIVERLGDYLI	22	0.684
4	8	QAYGD	5	0.602
85	91	GKKGPGG	7	0.588
		**t = 200 ns**		
1	7	MSWQAYG	7	0.808
47	66	ITGVMNDFNEPGSLAPTGLY	20	0.754
107	126	DEPMTPGQCNMIVERLGDYL	20	0.728
29	40	DGSVWAQSSTFP	12	0.624
76	80	QGEPG	5	0.522

Table S6. Conformational features of beta strands composition of Cor a 2 at different temperatures.

Temperature	% Strand	No. Beta sheet	No. Strands	Strand Conformation	No. Residues
300K	26.2	1	7	Ala22-Ile26<br>Val32-Gln35<br>Leu65-Tyr66<br>Lys71-Val74<br>Val82-Lys86<br>Gly91-Thr97<br>Ala100-Tyr106	34
350K	25.4	1	7	Ala22-Ile26<br>Ala34-Gln35<br>Leu65-Tyr66<br>Lys71-Ile75<br>Val82-Lys86<br>Gly90-Lys96<br>Leu101-Asp107	38
400K	26.2	1	6	Leu20-Gly27<br>Val32-Gln35<br>Met73-Ile75<br>Ile83-Lys86<br>Gly90-Lys96<br>Ala100-Asp107	34
450K	21.5	2	4	Ala23-Ile26<br>Ser31-Ala34<br>Gly91-Thr97<br>Ala100-Tyr106	22
			2	Leu65-Leu67<br>Tyr72-Val74	6
500K	15.4	1	4	Gly7-His10<br>Val82-Ile83<br>Val92-Thr97<br>Ala100-Asp107	20

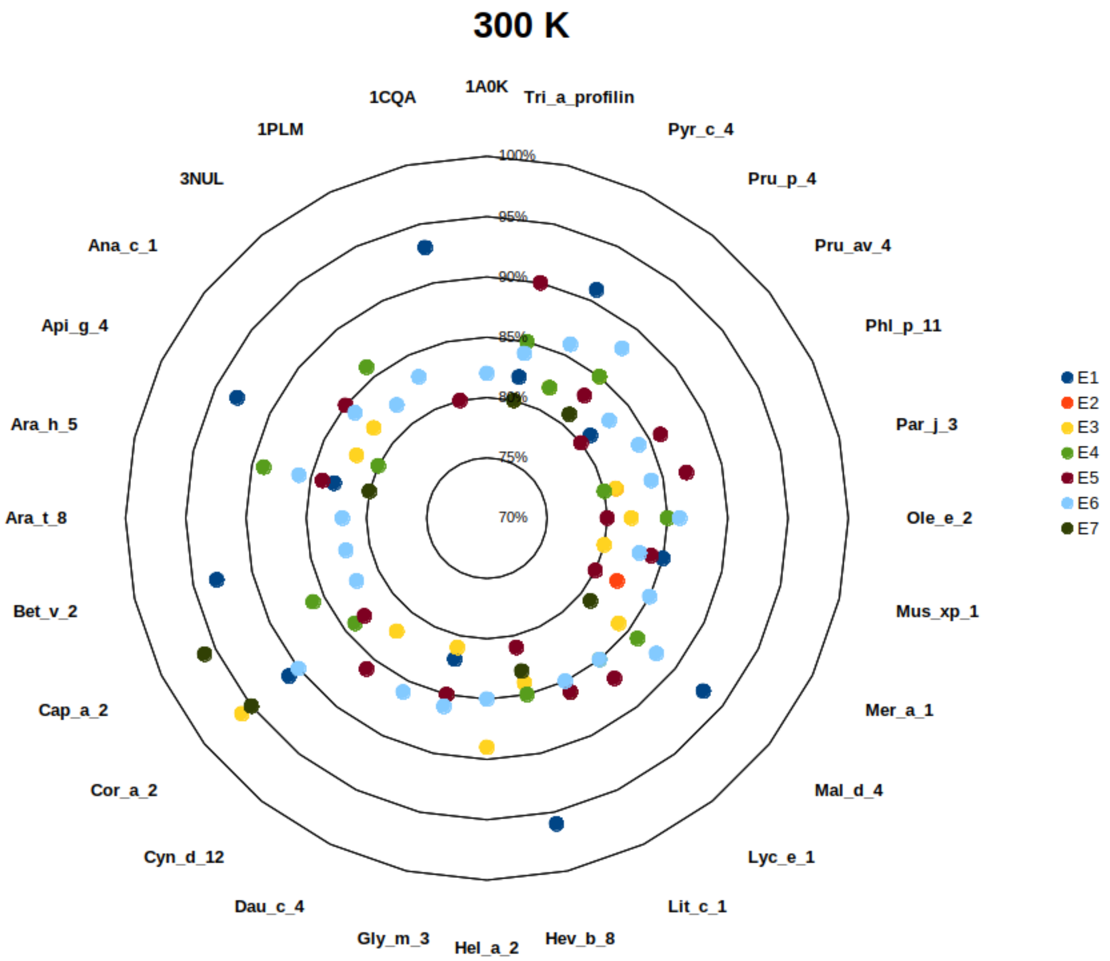

Figure S1. Cross-reactivity of profilin at 300K with allergens, the label E1 to E7 correspond for an epitope.

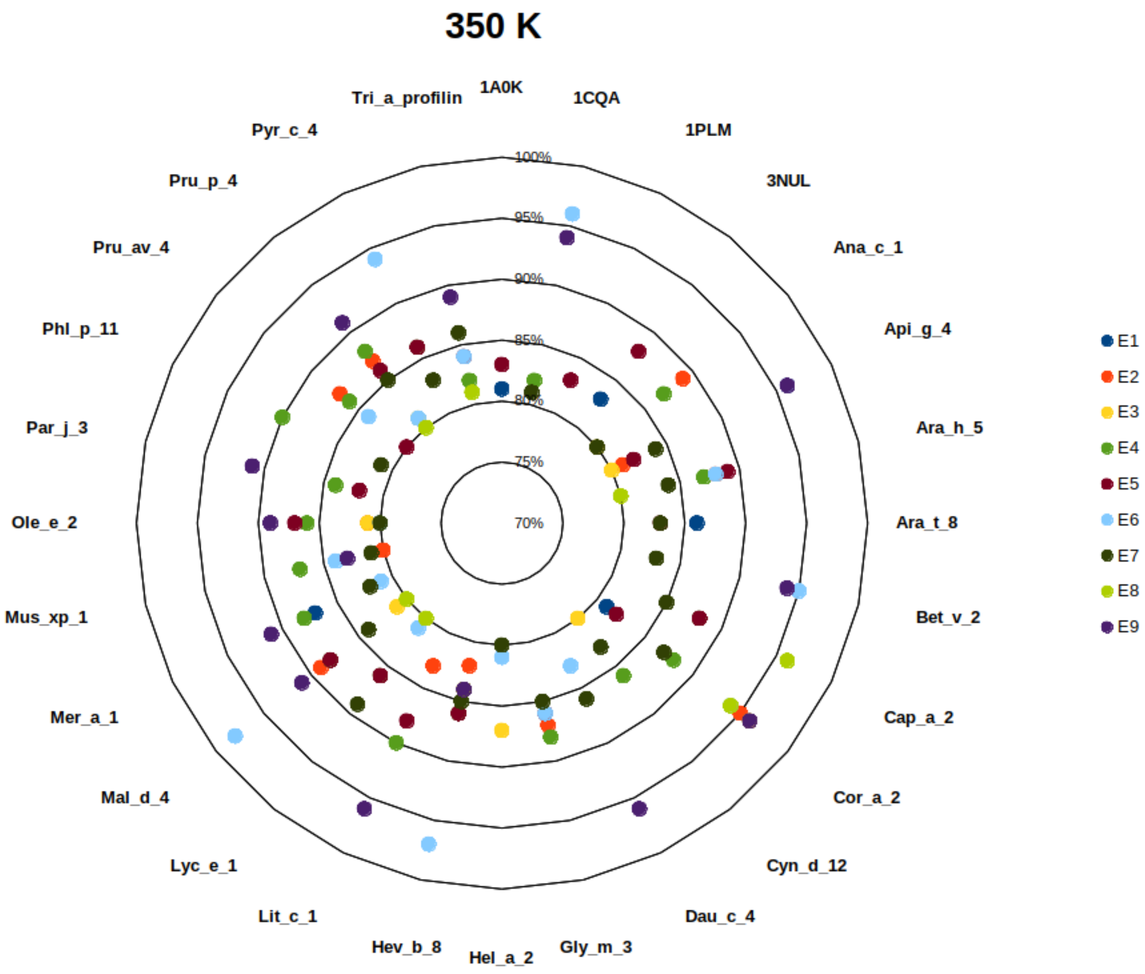

Figure S2. Cross-reactivity of profilin at 350K with allergens, the label E1 to E9 correspond for an epitope.

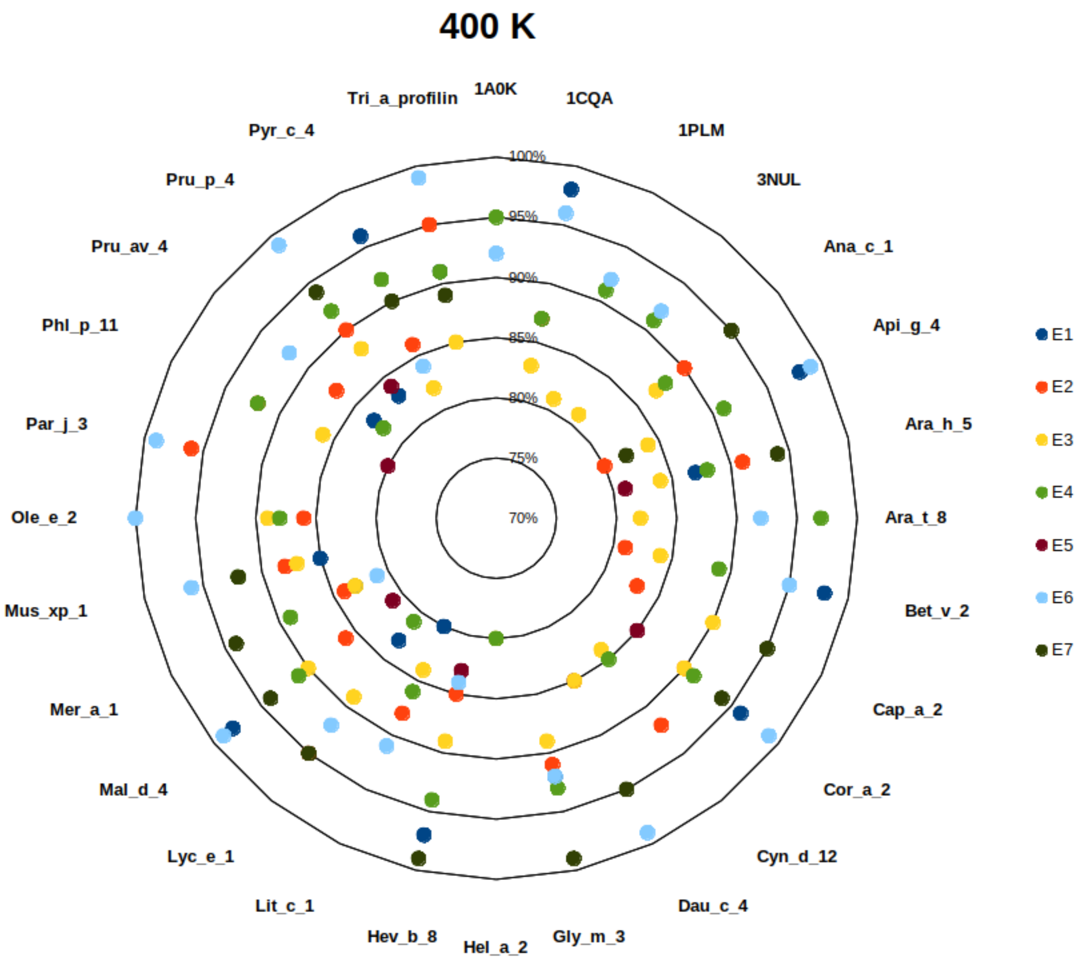

Figure S3. Cross-reactivity of profilin at 400K with allergens, the label E1 to E7 correspond for an epitope.

4

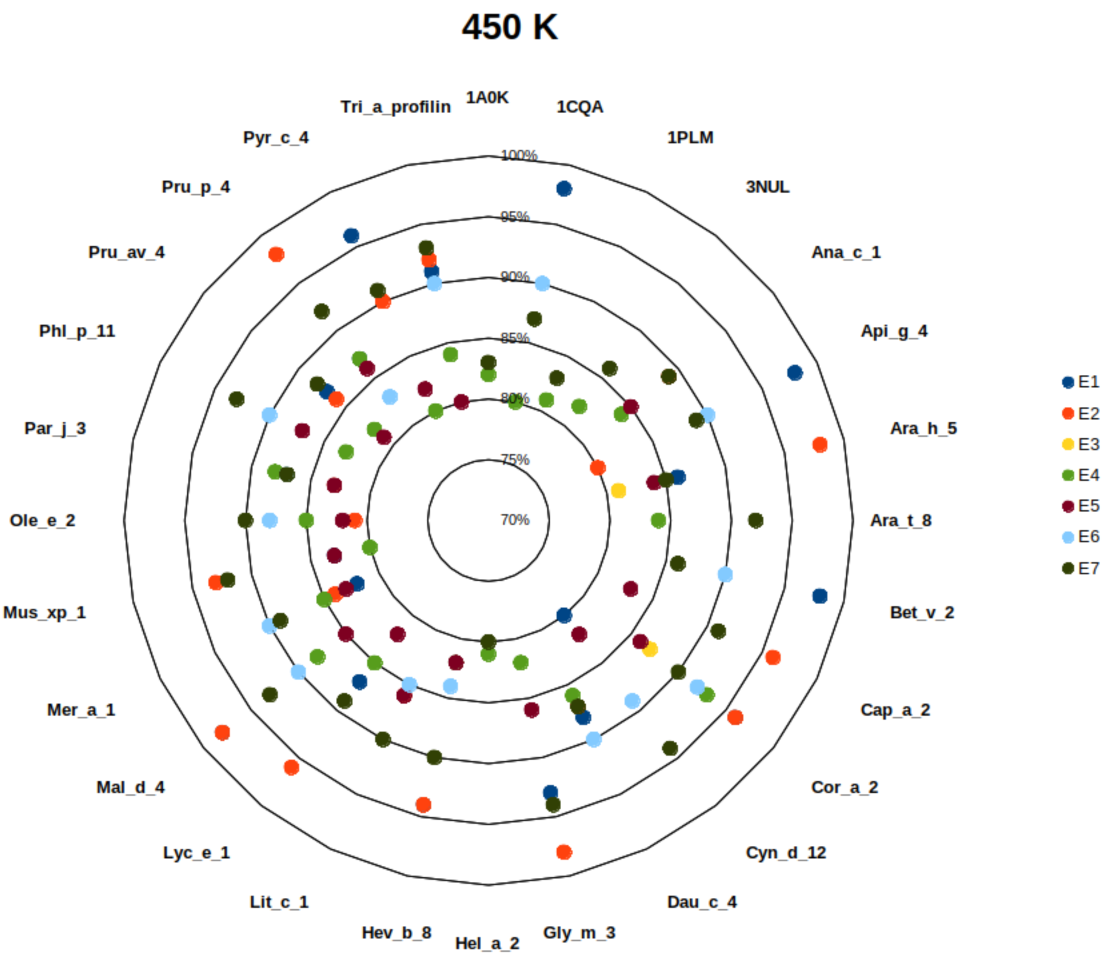

Figure S4. Cross-reactivity of profilin at 450K with allergens, the label E1 to E7 correspond for an epitope.

5

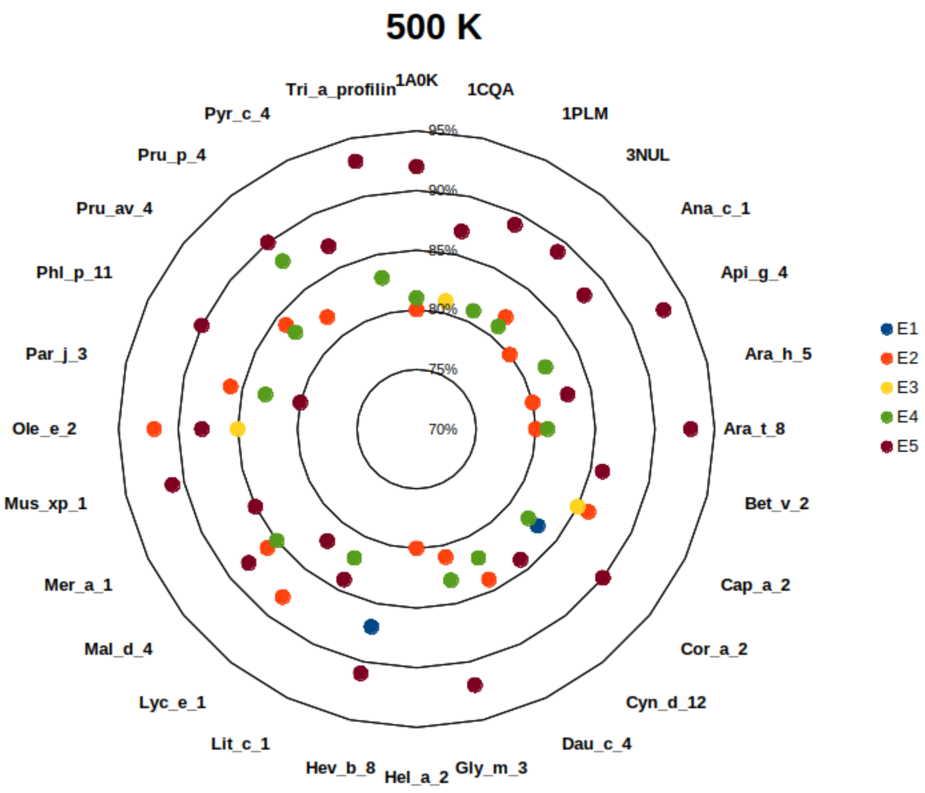

Figure S5. Cross-reactivity of profilin at 500K with allergens, the label E1 to E5 correspond for an epitope.
